# RELAX (REducing Levels of AnXiety): a study protocol for a parallel two-arm randomised controlled trial evaluating a web-based early intervention for pregnant women with high levels of repetitive negative thinking to prevent escalating anxiety during pregnancy and after birth

**DOI:** 10.1186/s13063-024-08516-9

**Published:** 2024-10-22

**Authors:** Brittannia Volkmer, Yogini Sawjani, Mary Newburn, Jo Bennett, Megan McGovern, Laura Bridle, Nathalie Towner, Laura McCabe, Katherine Clark, Sophie Webster, Alison Hylton-Potts, Lucy Mayer, Gertrude Senevirante, Debra Bick, Jill Newby, Kimberley Goldsmith, Michelle L. Moulds, Colette Hirsch

**Affiliations:** 1 Institute of Psychiatry, Psychology and Neuroscience, King’s College London, De Crespigny Park, London, UK; 2 Department of Women’s and Children’s Health, Guy’s and St Thomas’ NHS Foundation Trust, c/o, North Wing, St Thomas’ Hospital, 10 Floor Lambeth Palace Road, London, UK; 3 Department of Biostatistics & Health Informatics, Institute of Psychiatry, Psychology and Neuroscience, King’s College London, De Crespigny Park, London, UK; 4 King’s College Hospital NHS Foundation Trust, Denmark Hill, London, UK; 5 Epsom and St Helier University Hospitals NHS Trust, Wrythe Lane, London, UK; 6 Mid Cheshire Hospitals NHS Foundation Trust, C/O Leighton Hospital, Middlewich Road, Crewe, UK; 7 South London and Maudsley NHS Foundation Trust, Maudsley Hospital, Denmark Hill, London, UK; 8 Warwick Clinical Trials Unit, Warwick Medical School, Warwick, UK; 9 Black Dog Institute, Randwick, Australia; 10 School of Psychology, Faculty of Science, UNSW Sydney, Kensington, Australia

**Keywords:** Anxiety, Repetitive negative thinking (RNT), Perinatal mental health, Digital therapy, Cognitive bias modification (CBM), Prevention, Pregnancy

## Abstract

**Background:**

Perinatal anxiety is common: up to 40% of pregnant women and new mothers experience high levels of anxiety. Given its prevalence, interventions that are low-intensity, highly accessible and cost-efficient, and target modifiable risk factors for anxiety are needed. Repetitive negative thinking (RNT)—such as worrying about ways things will go wrong in the future or ruminating about past negative events—is a risk factor for the development of anxiety. RNT is maintained by the tendency to generate negative interpretations of ambiguous situations.

**Methods:**

A parallel two-arm randomised controlled trial will assess the efficacy of adding interpretation training (RELAX) to usual maternity care. Participants (*N* = 268) will be randomised to (i) 12 sessions of online interpretation training (RELAX) plus usual care, or (ii) usual care alone. We will assess anxiety, depression, RNT, and work and social adjustment at baseline, plus 4, 8 and 36 weeks later.

**Discussion:**

Should the intervention result in lower levels of anxiety than usual care, it could be an accessible, cost-effective way to help women who are vulnerable to experiencing anxiety in the perinatal period.

**Trial registration:**

ISRCTN 12754931. Registered 25th May 2023, prior to recruitment.

## Administrative information

Note: the numbers in curly brackets in this protocol refer to SPIRIT checklist item numbers. The order of the items has been modified to group similar items (see http://www.equator-network.org/reporting-guidelines/spirit-2013-statement-defining-standard-protocol-items-for-clinical-trials/).

**Table:** 

Title {1}	RELAX – REducing Levels of AnXiety—in pregnancy and after birth. A study to see if a new online training (RELAX) can reduce anxiety in pregnant women and new mothers
Trial registration {2a and 2b}.	ISRCTN 12754931. Registered 25th May 2023.
Protocol version {3}	The current protocol version is 1.9, dated 18.01.2024
Funding {4}	This study is funded by the NIHR Efficacy and Mechanism Evaluation Programme (NIHR131161), an MRC and NIHR partnership. The views expressed are those of the author(s) and not necessarily those of the NIHR or the Department of Health and Social Care.
Author details {5a}	SPIRIT guidance: Affiliations of protocol contributors. 1 Institute of Psychiatry, Psychology and Neuroscience, King’s College London, De Crespigny Park, London UK 2 Guy’s and St Thomas’ NHS Foundation Trust, c/o Department of Women’s and Children’s Health, 10th Floor, North Wing, St Thomas’ Hospital, Lambeth Palace Road, London UK 3 Department of Biostatistics & Health Informatics, Institute of Psychiatry, Psychology and Neuroscience, King’s College London, De Crespigny Park, London UK 4 King’s College Hospital NHS Foundation Trust, Denmark Hill, London UK 5 Epsom and St Helier University Hospitals NHS Trust, Wrythe Lane, London UK 6 Mid Cheshire Hospitals NHS Foundation Trust, c/o Leighton Hospital, Middlewich Road, Crewe UK 7 South London and Maudsley NHS Foundation Trust, Maudsley Hospital, Denmark Hill, London UK 8 Warwick Clinical Trials Unit, Warwick Medical School, Warwick UK 9 Black Dog Institute, Randwick, Australia 10 School of Psychology, Faculty of Science, UNSW Sydney, Kensington, Australia
Name and contact information for the trial sponsor {5b}	Lead sponsor: King’s College London, 16 De Crespigny Park, London, SE5 8AF Co-sponsor: Guy’s & St Thomas’ NHS Foundation Trust, R&D Department, 16th Floor, Tower Wing, Great Maze Pond, London, SE1 9RT
Role of sponsor {5c}	Study sponsors approved the study protocol. The funder (NIHR) will approve the main trial paper prior to submission.

## Introduction

### Background and rationale {6a}

Anxiety is common in the perinatal period, with up to 40% of pregnant women, and new mothers reporting high levels of anxiety [1]. Perinatal anxiety is associated with multiple significant adverse consequences, including reduced maternal responsivity to infants [2], impairments in childhood development, and a twofold increase in risk of a child developing psychological disorders [3]. Despite its documented prevalence and consequences, perinatal anxiety has received minimal research attention to date (in particular, relative to postnatal depression; [4, 5]). A limited number of treatment trials have evaluated psychological interventions for perinatal anxiety (see [6–9] for reviews) and have typically included women with high levels of anxiety or diagnosed anxiety disorders in the antenatal [10–13] and postnatal [11, 14] periods. Whilst it is no doubt clinically important to treat perinatal anxiety once it has emerged, it is equally important to explore the potential of early interventions targeting established risk factors to prevent or mitigate the development of anxiety symptoms later in the perinatal period.

Of the studies that have explored prevention in at-risk groups, most have tested multicomponent cognitive behavioural therapy (CBT) packages [15]. A limitation of this approach is that it precludes determining the specific treatment component/s that are most effective and deliver the most clinical benefit. In addition, these studies target cognitive and behavioural processes that play a role in psychopathology generally, rather than processes that have been established as predicting and maintaining psychological distress in the perinatal period specifically. Accordingly, an alternative approach is to target one identified, modifiable risk factor that predicts and maintains perinatal anxiety. Furthermore, interventions that target individuals who are identified as being at-risk of psychological problems are more effective than non-targeted universal approaches. Targeted preventative online interventions in particular are simpler to disseminate and deliver at scale. Moreover, online interventions such as LENS do not necessitate delivery by clinicians with specialist CBT training. Given this, there is a clear need for efficacious, evidence-based early-interventions which target known modifiable psychological risk factors which confer vulnerability to perinatal anxiety.

The tendency to engage in repetitive negative thinking (RNT)—worrying about the future and ruminating about the past—is an established risk factor for anxiety. Levels of RNT about a stressful life-event predict subsequent anxiety symptoms [16]. Pregnancy, birth, and adjustment to motherhood are potentially stressful and often characterised by uncertainty, and thus have the potential both to trigger and serve as a focus of RNT. Consistent with this, we have demonstrated a link between perinatal RNT and anxiety in pregnant [17] and postnatal [18] women. Our recent review [19] highlighted consistent evidence of associations between RNT and both depression and anxiety in pregnancy and postpartum. Moreover, worry in pregnancy was a consistent predictor of later (i.e. antenatal and postnatal) anxiety and depression [19].

No study to date has tested the possibility that an early intervention offered to those with RNT in pregnancy could result in lower anxiety later in the perinatal period. This is surprising given that we have effective interventions for RNT in the general population preventing the onset of later psychopathology. For example, Topper et al. [20] found that an intervention targeting RNT in young adults who reported high levels of RNT (i.e. Rumination-focused CBT [21]) resulted in less anxiety 12 months later. As a modifiable risk factor that predicts perinatal psychological distress, we propose that similarly targeting RNT early in pregnancy may result in less escalation of anxiety over the perinatal period. Such an intervention would need to be tailored to the unique experiences and challenges of pregnant women.

There is evidence that RNT is linked to a negative interpretation bias; that is, the tendency to interpret unclear or ambiguous information in a negative (rather than positive or benign) manner. A negative interpretation bias is a transdiagnostic process characteristic of individuals with a range of emotional disorders [22]. Hirsch and Mathews’ [23] cognitive model of RNT proposes that interpretation bias maintains RNT. In accord with this, there is evidence that RNT is associated with interpretation bias in non-pregnant clinical and non-clinical populations [24], and also in pregnant women [17]. Given the ambiguous nature of stressful situations during pregnancy (e.g. “will the scan show that my baby is ok?”), there are frequent opportunities to make negative interpretations (e.g. “the scan will find something wrong”) that fuel RNT, in turn escalating anxiety.

Cognitive bias modification (CBM) was initially developed as an experimental paradigm to investigate the downstream impact of manipulating cognitive biases (e.g. in interpretation, attention) on psychological symptoms. CBM has subsequently been employed to train individuals from populations characterised by unhelpful biases to adopt more adaptive alternatives (e.g. CBM to train positive interpretations of ambiguous stimuli, known as CBM-I), thus exploring its clinical utility. Hirsch et al. [25] found that 10 sessions of CBM-I which trained positive interpretations, compared to an active control condition, reduced both worry and anxiety at 1 month follow-up in people with a diagnosis of generalised anxiety disorder (GAD), compared to an active control condition. Similar findings emerged in a study with a community sample with high RNT (worry and/or rumination): participants who received CBM-I reported lower anxiety, depression and RNT at 1-month follow-up, relative to an active control condition [26], particularly when interpretation training incorporated imagery of the positive outcomes. In a GAD sample, Hirsch et al. [27] evaluated the effectiveness of the imagery enhanced multi-session CBM-I training delivered fully online (i.e. with no face-to-face researcher contact) relative to a control condition, and replicated these effects at 3-month follow-up. In addition, they reported high compliance with the intervention and excellent retention rates for follow-up assessments. Hirsch et al. [27] also demonstrated that the purported mechanism of change—interpretation bias—was effectively targeted by the intervention, and that the impact of CBM-I on reducing interpretation bias mediated effects on anxiety and worry.

Whilst these initial findings were promising, given the unique circumstances of the perinatal period (e.g. profound physical, hormonal, emotional and social changes), we could not assume that the mechanisms underpinning RNT (nor the interventions that address them) are the same in pregnant and non-perinatal samples. Accordingly, we conducted a proof-of-principle study [28] to examine whether a single session of CBM-I could effectively modify interpretation bias in pregnant women (≥ 16 weeks gestation) with high levels of worry. Relative to participants in the active control condition (in which the ambiguous scenarios were also presented but remained unresolved), those who received CBM-I generated more positive interpretations and reported fewer thought intrusions (on a behavioural worry measure). Thus, CBM-I effectively induced a positive interpretation bias and resulted in less negative thought intrusions (a proxy for worry) in pregnant women who were high worriers, raising the possibility of its capacity as an intervention to target RNT in the perinatal period.

To increase the accessibility and uptake of psychological treatment in the perinatal period, research has focused on the development and evaluation of online interventions [29, 30]. Online interventions have the potential to increase access to psychological interventions for expectant and new mothers, for whom there are likely to be multiple practical barriers to attending therapy in a clinical setting. Moreover, they also reduce the burden on typically over-stretched perinatal mental health services, leaving capacity available for those who need specialist input. Indeed, our extensive Patient and Public Involvement and Engagement (PPIE) work indicates that interventions during pregnancy, rather than post-birth are favoured, given that women would have more time to complete our brief (20-min sessions) when pregnant, rather than when they are adapting to the challenges and responsibilities associated with caring for a newborn and are sleep deprived. They also felt that an online intervention that they could complete when and where works for them would be particularly helpful since it would negate the costs of travel and provide flexibility around work, childcare and other responsibilities. They also mentioned that they would like to hear about the intervention via both NHS services and non-NHS routes, such as third sector organisations that support women over the perinatal period or social media. The development of a low intensity (self-help), highly accessible, cost-effective intervention to address perinatal anxiety is an exciting prospect. Not only could such an intervention lead to fewer anxiety symptoms in pregnant women with an established risk factor (RNT), it also has the potential to reduce the need for specialist, costly, high-intensity mental health support in the postnatal period (e.g. individual cognitive behaviour therapy).

### Objectives {7}

We will conduct a parallel two-arm randomised controlled trial to establish the efficacy of a multi-session, web-based CBM-I intervention tailored to women in the perinatal period (RELAX) in reducing anxiety in pregnancy and postpartum compared to usual care (UC) alone. We hypothesise that pregnant women with high levels of RNT who complete RELAX (plus UC) will report lower levels of anxiety during pregnancy and after birth relative to pregnant women who receive just UC. We will focus on the key cognitive mechanism of testing whether interpretation bias mediates treatment effects, and whether interpretation bias prevents anxiety.

### Trial design {8}

A two-arm, parallel-group, multi-site, superiority, Phase 2b randomised controlled trial in which randomisation will be stratified by recruitment site, parity, and pregnancy complications. Pregnant women (16–28 weeks gestation) with high levels of RNT (RTQ-10 score ≥ 28) and up to a moderate level of anxiety (GAD-7 score < 15) will be randomly allocated to one of two conditions. Specifically, they will either be assigned to either the RELAX intervention in which they will complete 12 web-based Cognitive Bias Modification for Interpretation (CBM-I) training sessions in a 4 week period alongside their usual maternity care or will continue with their usual maternity care.

## Methods: participants, interventions and outcomes

### Study setting {9}

The intervention is being delivered online via the REducing Levels of AnXiety (RELAX) platform. The RELAX platform [31] was built and is hosted by Avegen, a digital healthcare company who deploy digital health applications through cloud-hosted, product development platforms [32].

### Eligibility criteria {10}

Inclusion criteria are as follows: pregnant women (16–28 weeks gestation); high level of self-reported RNT (RTQ-10 (trait) ≥ 28; [33–35]; up to only a moderate level of anxiety (GAD-7 < 15); aged ≥ 18 years; living in the UK; able to understand oral and written English; normal or corrected-to-normal hearing and vision; access to the internet on a PC, laptop or tablet (other than mobile phone); provision of an email address and phone number (for contact with the team). Initial eligibility is based on participant self-reported responses at screening.

Exclusion criteria are as follows: reporting symptoms consistent with a current psychiatric diagnosis (assessed via the clinical interview schedule revised (CIS-R); [36] at screening; current or past diagnosis of a psychotic disorder (e.g. schizophrenia), eating disorder, substance use disorder (e.g. alcohol dependence) and/or personality disorder (e.g. borderline personality disorder); current or recent history of risk (e.g. suicidal thoughts on the PHQ-9, item 9 > 1); suicide attempt within the past 2 years and/or self-harm within the last year; history of stillbirth, neonatal death, or multiple (i.e. ≥ 3) miscarriages; current participation in another study evaluating a treatment for a mental health problem; not being registered with a GP in the UK.

We use the term “women” to refer to those who are pregnant. We acknowledge that not all people who are pregnant and give birth identify as women, and it is important that evidence-based care for maternity, perinatal and postnatal health is inclusive. The study is open to anyone who is currently pregnant however they choose to identify, and we welcome all those who are eligible.

### Who will take informed consent? {26a}

Participants provide informed consent at the start of the baseline assessment and their electronically signed informed consent form will be submitted via the online RELAX platform.

#### Eligibility assessment and study outline call

Pregnant women interested in taking part will be able to register on the RELAX platform, where they can also access the Participant Information Sheet. Individuals who register and provide consent for screening will complete a series of validated questionnaires along with additional questions assessing their eligibility against the inclusion/exclusion criteria. Individuals will be asked to provide their full name, age, sex at birth, email address, phone number, GP, midwife and maternity service details, and indicate where they heard about the study. They will also be asked their number of weeks gestation, along with questions regarding their pathway of care, current/past pregnancy complications and parity (i.e. whether they have had previous live births).

Individuals will be notified that they are not eligible at the point at which they provide an answer to a question that renders them ineligible, i.e. they will not be asked to complete any further items in the screening survey. Individuals who are eligible after completion of the screening questionnaire will be emailed by the team to arrange a telephone call (i.e. study outline call).

The study outline call will take approximately 20 min. The researcher will provide more information about the study (e.g. outline time commitments, explain randomisation), verify details provided in the screening questionnaire and answer any questions. The presence of current suicidal thoughts is assessed on the study call, and a risk assessment is conducted if needed and appropriate action in keeping with the risk protocol will involve a clinician if appropriate, or additional signposting to support services. A risk assessment is also conducted if the potential participant marked “1” indicating “Several days” to item 9 “Thoughts that you would be better off dead or hurting yourself in some way” on the Patient Health Questionnaire (PHQ-9) at screening [37]. In the event that a participant indicates during the call that they are unable to commit to the study and/or complete the RELAX sessions under the timeframe/conditions specified (e.g. they do not have time to complete three sessions per week, are unable to generate mental images, or do not have access to a quiet space), they will be offered the option to not take part (if still potentially eligible) and will be reimbursed with a £5 voucher for their time. If at the end of the call the participant is eligible and willing to continue, they will be provided with information about completing the baseline (T0) assessment and sent the link to do this.

#### Baseline assessment

Participants will complete the baseline (T0) assessment via the online RELAX platform, within 10 days of their screening questionnaire, after which time the link will expire. Any participant who has not completed the assessment by that time will no longer be able to do so.^1^ As part of the baseline assessment, participants will first provide informed consent and then proceed to complete the baseline measures:

Demographic variables: date of birth, ethnicity, religion, relationship status, social support, highest level of education, employment status, pre-existing health condition, number of children, estimated due date.

The *Repetitive Thinking Questionnaire (RTQ-10 (trait))* [33] is a 10-item measure of trait RNT. Participants rate the extent to which each item (e.g. “I have thoughts or images about all my shortcomings, failings, faults, mistakes”) is true for them when they are distressed or upset. The highest score possible is 50, indicating the greatest level of RNT. The measure demonstrated high internal consistency in a clinical sample of individuals diagnosed with major depressive disorder, social phobia, GAD or dysthymia (*α* = 0.92, mean inter-item correlation = 0.53 [33], Cronbach’s *α* = 0.90 [28]).

The *Generalised Anxiety Disorder Questionnaire (GAD-7)* [38] is a 7-item measure of anxiety symptoms over the past 2 weeks (e.g. “Feelingnervous, anxious or on edge?”). Items are scored 0–3 where 0 as “not at all,” 1 = “several days,” 2 = “more than half the days” and 3 = “nearly every day.” The GAD-7 has good test–retest reliability (intraclass correlation = 0.83) [38], Cronbach’s *α* = 0.87 [28]).

The *Patient Health Questionnaire (PHQ-9)* [37] measures depressive symptoms over the past 2 weeks. Item ratings are summed to produce a total score from 0 to 27. Participants who score above 1 on item 9: “Thoughts that you would be better off dead or hurting yourself in some way” will not be eligible for inclusion in the trial. The PHQ-9 is a reliable and valid measure of depression severity (*α* = 0.86 in an Obstetrics-Gynaecology population [37], Cronbach’s *α* = 0.84 [28]).

The *Penn State Worry Questionnaire (PSWQ)* [39] measures worry (e.g. “My worries overwhelm me”) and participants rate each statement on a scale of 1 (“not at all typical of me”) to 5 (“very typical of me”). Test–retest reliability is high [39], Cronbach’s *α* = 0.83 [17].

The *Work and Social Adjustment Scale (WSAS)* [40] is a five-item measure of impaired functioning [40]. Item 1 assesses impact on ability to work, item 2 assesses impact on home management, item 3 assesses impact on social leisure activities, item 4 assesses impact on private leisure activities and item 5 assesses impact on close relationships. Scores range from 0 to 40, with high scores indicating more impaired work and social functioning. We modified the measure to make it possible for participants to mark N/A to item 1 which relates to work, and plan to prorate this item with an average score when analysing the data in keeping with clinical practice. Test–retest reliability correlation = 0.73, internal scale consistency ranged from *α* = 0.70–0.94 [40].

The *Perinatal Anxiety Screening Scale (PASS)* [41] measures anxiety in antenatal and postpartum women and contains 31 items. Participants rate how often they experience each item (e.g. “Fear that harm will come to the baby”) in the past month. Item ratings are summed to produce a total score between 0 and 93, with higher scores indicating greater levels of perinatal anxiety. Test–retest reliability for the PASS is good (correlation for global scores 0.74 [41], Cronbach’s *α* = 0.94 [28]).

The *Edinburgh Postnatal Depression Scale (EPDS)* [42] is a 10-item measure used to assess depression symptoms in pregnant participants. Higher score indicates more severe perinatal depression. The EPDS high test–retest reliability for total scores (ICC = 0.92) [43]. Cronbach’s *α* = 0.84 [28]).

The *Recognition Test* [27, 44] will be completed at baseline and T1 to measure interpretation bias. It has been adapted from previous research [25, 27, 28, 44] for the purpose of this trial, and contains 12 scenarios (half are pregnancy-related, and the remaining scenarios are non-pregnancy related). The trial team and Research Midwife checked all pregnancy scenarios to ensure they were up-to-date, relevant and factually correct.

In the first part of this task participants are presented with a title and ambiguous scenarios. The final word in each scenario is presented as a word fragment with missing letters. Participants are instructed to enter the first missing letter of the word fragment. In the second part of the task, participants are presented with the title of the scenario and a series of four statements. They are asked to rate the degree to which each statement is similar in meaning to the original scenario on a 4-point scale (i.e. very different, fairly different, fairly similar, or very similar in meaning). Two of the statements represent potential interpretations of the ambiguity. One is a positive target relating to a positive interpretation of the scenario, the other a negative target related to a negative interpretation of the scenario. The remaining two statements are unrelated to the scenario but are either positive or negative in valence. Higher similarity ratings for positive targets indicate a more positive interpretation of that scenario. An example of a pregnancy scenario is as follows:

##### Midwife diet advice

You are pregnant and meeting with your midwife. They ask you how you have been managing your diet. You mention to them that you have not been eating very well as sickness has put you off certain foods or large meals. From the look on their face, you can tell what they are going to say about your diet.

Has sickness put you off large meals? (Yes/No).

Positive target: Your midwife is understanding and suggests that the way you are managing your diet is fine.

Negative target: Your midwife is unsympathetic and stresses how important maintaining a healthy diet is for the baby.

Positive foil: Your midwife provides you with some useful advice for dealing with morning sickness.

Negative foil: Your midwife repeats something you have already mentioned and you get the feeling that they are not listening to what you have to say.

Mean scores for both positive and negative targets are calculated and an interpretation bias score is generated by subtracting mean positive targets from mean negative targets. There are two sets of ambiguous scenarios which are presented in counterbalanced order across T0 and T1 assessments across participants.

*Psychological contact and treatment form* (*past diagnosis of a mental health condition, current psychological treatment and mental health medication receipt, previous contact with a health professional in relation to your mental health).*

### Additional consent provisions for collection and use of participant data and biological specimens {26b}

Not applicable, this trial does not collect biological specimens.

## Interventions

### Explanation for the choice of comparators {6b}

On completion of the baseline assessment, participants will be randomised to either the RELAX intervention alongside UC or UC alone. The research team will send a letter via NHS.Net email, containing details about the trial and outlining the participant’s involvement to their GP and midwife/maternity service. For participants recruited outside of a participating NHS site (e.g. via social media), the midwife/maternity services letter will be given to the participant to pass on to their midwife. For participants at the NHS recruitment sites, their participation will be recorded in the electronic clinical notes.

### Intervention description {11a}

#### Active arm – RELAX + usual care (UC)

Participants in the active arm will complete 12 sessions (approximately 15–20 min duration) of the RELAX intervention via the online platform using a computer, laptop or tablet, in the 4 weeks post-randomisation. Participants will be asked to complete their first session within 24 h of the baseline assessment. Participants will be able to choose when to complete the remaining sessions, although instructions on the platform will encourage them to complete 3 sessions per week. Participants will not be able to complete more than one session per day (unless they completed part of a session the previous day, in which case they can complete that session the following day alongside a new session).

#### Usual care (UC)

Usual care typically involves monitoring by maternity services and contact with a health visitor. Women may also be offered information on self-referral to local psychology services, where they will typically be put on a wait list or be offered generic interventions (e.g. group treatment, computerised CBT). Those in the control arm will only receive usual care and when they log onto the RELAX platform after T0, they will only see information regarding their next assessment, which will become active for completion on the date they are scheduled to open.

#### RELAX session description

The sessions involve listening to 30 pre-recorded audio descriptions of ambiguous everyday situations (scenarios) that can be interpreted in both negative and positive ways and are pertinent to the daily lives of pregnant women. Approximately 50% of the scenarios describe situations that occur in pregnancy, e.g. attending medical appointments, managing pregnancy symptoms, preparing for baby and the transition to motherhood.

The scenarios were developed with feedback and input from women with lived experience of perinatal anxiety, via focus groups and individual interviews. In addition, we drew on themes of worries and anxiety commonly reported during pregnancy, identified in the research literature [18]. All scenarios were developed by trained and expert research team members (BV, CH, MM, YS), and a proportion of the scenarios checked for suitability and clinical accuracy by both the RELAX PPIE group and midwives in the wider research team.

Worry scenarios tend to focus on worry about the future, whilst rumination scenarios tend to focus on events from the past. Across the 30 scenarios presented per session, approximately 60% are worry focussed and 40% rumination focussed. In order to provide a mix of scenarios per session, half of the scenarios were not pregnancy related but were carefully selected for suitability for pregnant women and drawn from scenarios used Hirsch et al. [27].

The scenarios are all ambiguous and involve uncertainty. The ambiguity can be resolved in either a positive or negative manner. The scenarios are designed such that the ambiguity is either resolved for the participant with a positive interpretation provided (positive scenarios), or the ambiguity is left unresolved (ambiguous scenarios), and the participant is required to generate a positive outcome for the situation. Positive scenarios are always presented earlier in the session than ambiguous ones. As the sessions progress, participants are presented with increasing numbers of ambiguous scenarios; 50% in sessions 1–4, 70% sessions 5–8 and 90% sessions 9–12.^2^ Furthermore, in sessions 1 to 5, some positive scenarios are presented again but in their ambiguous form, in order to help participants to generate a positive interpretation themselves (examples provided in Table 1).

**Table 1:** Examples of RELAX ambiguous intervention scenarios

Positively resolved scenario	Comprehension question
You are at a routine ultrasound scan. It is taking longer than you expected. The sonographer is very quiet, but from her face you know she thinks things are fine	Do you think that the sonographer has found a problem?
You are going to visit a friend for the weekend but are thinking about cancelling as you have not been feeling great recently. You decide to still go as you don’t want to let your friend down. On the journey back home, you reflect on the weekend and realise visiting your friend was worthwhile	Do you regret going to visit your friend?
You realise how many things you have left to prepare for the arrival of your baby and decide to write down things you can do this weekend to make a start. Doing this makes you feel calmer	Was making a list of things to do a bad idea?
It is your birthday approaching, and the first birthday where you have been pregnant. As you think about being a year older you think about your current situation and are filled with a sense of satisfaction	Are you thinking positively about your birthday this year?
Ambiguous scenario	Comprehension question
You are at a routine ultrasound scan. It is taking longer than you expected. The sonographer is very quiet, but from her face you know what she is thinking	Will the sonographer tell you that there is a problem?
You are attending a pregnancy exercise class and you are following the class quite well this week. At a break there is a chance to talk to other women attending, who all seem to know each other. As you approach them, you can tell whether you will be included in the conversation	Was it hard to join in the conversation with the group?
You talk with a friend about what she did to connect with her baby during pregnancy. She enthusiastically gives you some ideas and you think over whether they will be helpful or not	Will your friend’s suggestions be useful for connecting with your baby?
A friend is talking to you about how she is feeling during her pregnancy, and you start dwelling on the fact that you have both have completely different symptoms. You wonder what that could mean about your pregnancy	Does it matter that you have different symptoms to your friend?

There are 30 trials per session (scenario, comprehension question and rating). For each trial the participant listens to the scenario and imagines themselves in positive outcome of the situation. Participants are then prompted to answer either yes or no to a subsequent comprehension question designed to reinforce the positive interpretation. Participants receive accuracy feedback with a tick or cross depending on accuracy of the response provided. For 50% of the trials, participants will then be asked to rate how they experienced the scenario on a visual analogue scale from 0 (not at all positive) to 100 (extremely positive). For the remaining 50% of trials, participants will be asked to rate how vividly they were able to imagine the ending on a scale from 0 (not at all vivid) to 100 (extremely vivid). Written feedback will be presented on the screen and will be dependent upon the ratings that participants provide (e.g. “Good effort!”, “That’s great!”).

#### Training to use the intervention

The first session will last approximately 30 min, whilst the remainder are 15–20 min duration.

In session 1, participants will be presented with brief videos that outline the rationale for training, and details about how to complete training sessions and how to schedule their sessions. Participants will be asked to complete the sessions in a space free of distractions, ideally using headphones to enable them to be immersed in the scenarios. To facilitate positive imagery generation, participants will complete a one-off imagery training. This will involve watching a short video about imagery and completing imagery exercises, as per Hirsch et al. [27]. Participants will then practice imagining positive outcomes of the scenarios and answering questions, after which they will complete the 30 trials. At the end of the first session, participants will complete expectancy ratings of how logical the intervention seems on a scale of 0 (not logical at all) to 4 (very logical) and how useful they expect it to be on a scale of 0 (not at all useful) to 4 (very useful).

Sessions 2 to 12 will comprise 30 trials and take between 15 and 20 min to complete. At the end of session 3, participants will be asked to notice when they are thinking negatively in day-to-day life and then try to identify a potential positive outcome for the situation. The goal of this is to help participants generalise the training into daily life. Participants will also be sent a text message the day after sessions 3, 6 and 9 to remind them to try and identify positive outcomes when they notice themselves worrying or thinking negatively about a situation.

A minimum of 20 telephone interviews will also be conducted with participants allocated to the RELAX condition. This nested qualitative study will include participants who complete all, some or none of the RELAX sessions. In addition, we will include participants who withdrew from the sessions in order to determine their views of the intervention and to provide them with an opportunity to suggest ways in which it could be improved.

### Criteria for discontinuing or modifying allocated interventions {11b}

Participants will be informed that they are free to withdraw from the trial completely or discontinue with their RELAX sessions at any time.

### Strategies to improve adherence to interventions {11c}

Alerts have been designed to automatically nudge participants if they have left a session incomplete, e.g. they will receive a reminder the next day and can be able to finish the session that day (and can complete their next full session that same day if they wish). Participants will also receive email reminders from the RELAX platform to complete sessions if they have not completed the target number of sessions by a particular time, e.g. if less than 3 sessions have been completed by the end of week 1. Researchers monitoring progress will contact participants via phone call, email or text message (SMS) if they are progressing through sessions too slowly, so that they will be able to complete them all within the required timescale. Researchers will help troubleshoot any issues participants may be experiencing regarding the scheduling of sessions or technical issues.

### Relevant concomitant care permitted or prohibited during the trial {11d}

Participants in both trial arms will continue to receive usual maternity care. They may be offered information about how to self-refer to local psychology services where they will be put on a waiting list or be offered generic interventions (e.g. group treatment, computerised CBT) or advised to contact their GP, who may signpost them to counselling services and / or discuss medication.

As usual care may potentially involve being offered psychological treatment, individuals who are currently receiving or have recently received psychological treatment will not be excluded from the trial. Engagement in current psychological interventions at any point during the trial will be monitored and information for this requested at each follow-up assessment. The RELAX intervention will not be available outside of the trial, so there is no risk of the UC arm being exposed to the intervention or other CBM-I interventions given that they are also not currently available in the UK.

### Provisions for post-trial care {30}d

There are no provisions for post-trial care as a part of this study.

### Outcomes {12}

#### Clinical outcomes

The primary outcome is the mean difference in anxiety (measured using GAD-7) at 8 weeks post-randomisation between arms, adjusting for anxiety at baseline. For secondary outcomes, we will measure the mean differences between arms in:

- Anxiety (GAD-7) at 36 weeks post-randomisation
- Depression (PHQ-9) at 8 weeks post-randomisation and 36 weeks post-randomisation
- RNT (RTQ-10 (trait)) at 8 weeks post-randomisation and 36 weeks post-randomisation
- Trait worry (PSWQ) at 8 weeks post-randomisation and 36 weeks post-randomisation
- Perinatal depression (EPDS) at 8 weeks post-randomisation and 36 weeks post-randomisation
- Perinatal anxiety (PASS) at 8 weeks post-randomisation and 36 weeks post-randomisation
- Work and social functioning (WSAS) at 8 weeks post-randomisation and 36 weeks post-randomisation

#### Mechanistic outcome

The primary mechanistic outcome is the mean difference in interpretation bias (measured using the Recognition Test) at 4-week post-randomisation between arms, adjusting for baseline interpretation bias. The mediating effect of the intervention on GAD-7 at T2 via interpretation bias at T1 will also be evaluated.

### Participant timeline {13}

**Fig. 1:**
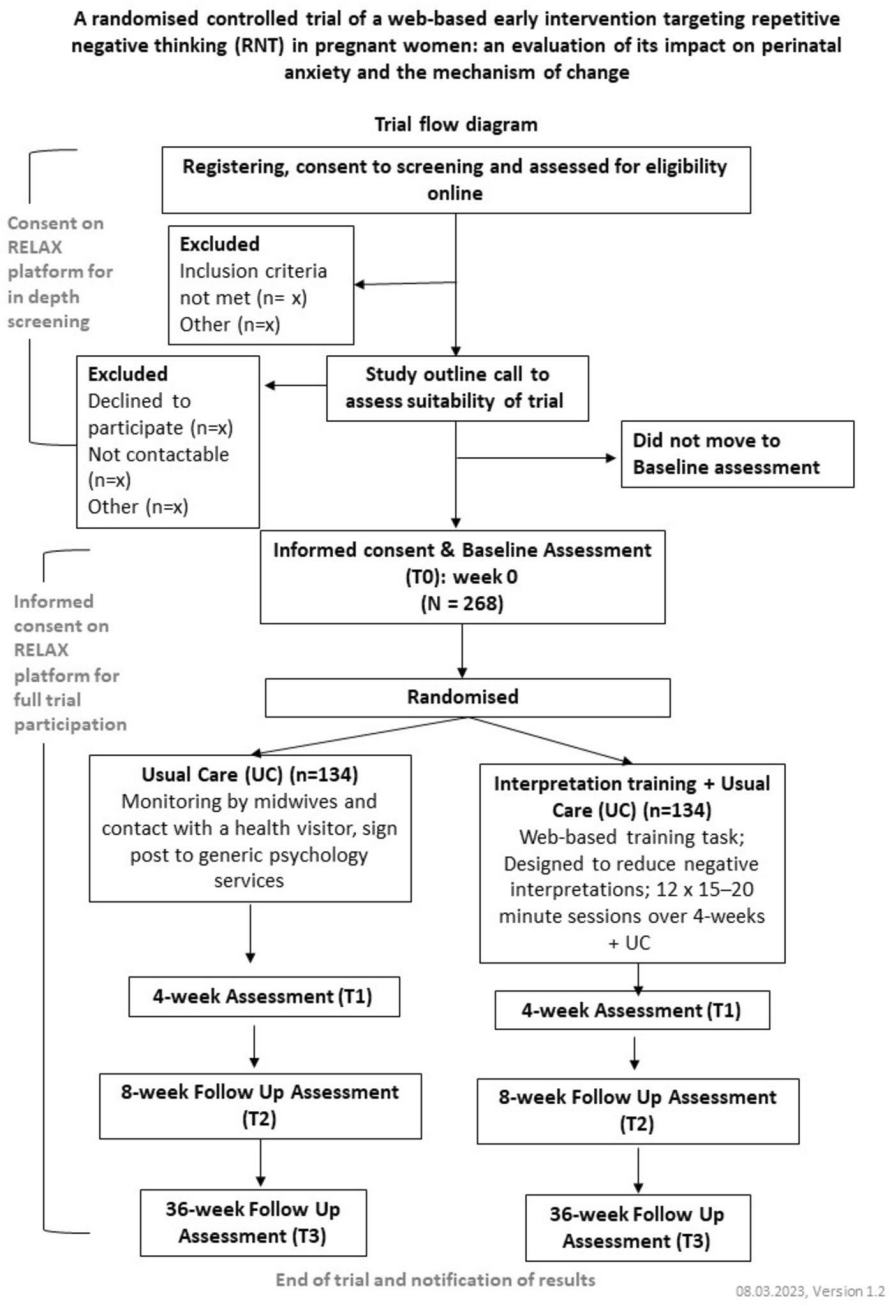
RELAX trial flow diagram

At the point of enrolment into the study all participants will be between 16 and 28 weeks pregnant. Participants will be enrolled in the study for approximately 9 months in total (Fig. 1). Participants in the intervention arm will have 4 weeks from the point of randomisation to complete the 12 RELAX sessions.

#### Follow-up assessments

There are 4 assessments yoked to randomisation that all participants are required to complete: baseline pre-randomisation (T0; 16–28 weeks gestation); 4 weeks post-randomisation (T1; end of intervention for active arm; 20–32 weeks gestation); 8 weeks post-randomisation (T2; 24–36 weeks gestation), and 36 weeks post-randomisation (T3; 12–24 weeks post-birth).

### Sample size {14}

#### Clinical efficacy sample size

For a two-sided independent samples *t*-test, alpha = 0.05, assuming one baseline and three post-randomisation measures with correlation rho = 0.5 (conservative based on baseline − T2 estimate from Hirsch, Krahe et al., 2021 [27] of rho = 0.42), deflation factor of 0.5 [45], and accounting for 35% attrition (based on online CBT: [46]), we will need 268 participants in total (134 per arm) to have 90% power to detect a GAD-7 effect size of 0.35 (powered slightly conservatively compared to T2 GAD-7 effect size of 0.40 from Hirsch, Krahe et al., 2021 [27]).

#### Mechanistic analysis sample size

Estimates of the mediation parameters from Hirsch, Krahe et al. [27] as follows: standardised estimate of the a path (effect of intervention on mediator) = 0.5, b path (effect of mediator on outcome) = 0.2, and c’ path (direct effect of intervention on outcome) = 0.15, providing parameters for Monte Carlo simulations with 10 K repetitions to calculate power in Mplus [47], a sample size of 268 would give > 99% power to detect the mechanistic action of the intervention on interpretation bias (a path), and 83% power to detect the indirect or mediated (a x b) effect of the intervention on GAD-7 via interpretation bias.

### Recruitment {15}

We expect that a higher proportion of participants will be recruited from the combined NHS sites. Particular site targets will be determined according to the timing of site opening. A minimum of one quarter of participants are expected to be recruited from the KCL route.

#### NHS sites

The study aims to recruit 268 participants. We will recruit participants through one of the four participating NHS hospital sites (Guy’s and St Thomas’ NHS Foundation Trust (GSTT), King’s College Hospital NHS Foundation Trust (KCH), Epsom and St Helier University Hospitals NHS Trust (ESTH) and Mid Cheshire Hospitals NHS Foundation Trust (MCHT)). Staff who are part of the potential participants’ direct care team will screen the medical records of women booked to receive antenatal care at each participating site to identify potentially eligible participants and notify them about the study. In addition, pregnant women will be approached in antenatal clinic waiting areas and during routine antenatal appointments and informed about the study, or given study details, such as the Participant Information Sheet or study flyers.

Posters advertising the study will be displayed in and around antenatal waiting areas and clinic rooms, utilising a QR code for ease of signposting to the RELAX platform. Flyers and business cards will also be utilised, dependent on local permission, for potential participants to take study details with them or leave their details so that they can be contacted by the RELAX research team.

At some of the NHS sites, research midwives (including “flexible” clinical research midwives) will also provide information on the study to potentially eligible women via email and phone, following screening of clinical records, and signpost them to the platform (website).

Other NHS recruitment initiatives will include providing details about the study during antenatal ward rounds and meetings, patient classes, and via research circulars and events. In addition, RELAX will be listed on NHS site-specific maternity apps seen by pregnant women, so that all pregnant women registered at the participating site will receive push notifications about the RELAX study.

#### Other recruitment routes

The research team at King’s College London (KCL), supported by PPIE advisers, partner organisations, charities, third sector organisations and affiliated communications teams will share information about the study via social media and community organisations. This will include advertising on channels such as Instagram [48], Facebook [49], and X [50], and approaching pregnancy support groups community organisations and charities and requesting that they promote the study in their venues, through events and on their social media platforms. This community and social media recruitment approach has been adopted using testimonies and community endorsement with the aim of recruiting a diverse range of participants, including those often disproportionately excluded from research, such as Black and Asian women. Participants recruited via these recruitment routes will be allocated to the “KCL” recruitment site strata. In addition, posters will also be displayed in antenatal waiting areas at non-participating NHS sites and health care facilities across the UK where permission has been given. A small number of GP practices in South London have also been invited to identify pregnant women who may be eligible for the trial and recruit participants as Participant Identification Centres (PIC) sites for the study. Participants recruited via these methods will also be allocated to the “KCL” recruitment site strata.

## Assignment of interventions: allocation

### Sequence generation {16a}

Stratified randomisation will be carried out via the RELAX platform by an embedded computer-generated algorithm developed by Avegen and embedded in the platform. Randomisation will be at the individual level, in a 1:1 ratio using randomly permuted blocks with varying block sizes. The algorithm selects the next appropriate allocation for an individual based on their stratification variable values.

Stratification is by.

- recruitment site (either one of the four participating NHS Trusts: GSTT v KCH v ESTH v MCHT) or KCL (pathway covering all other social media and community recruitment routes, including non-participating NHS sites recruitment, e.g. via posters or GP practices)
- parity (previous livebirths vs no previous livebirths)
- previous pregnancy complications (no pregnancy complications vs some pregnancy complications (past or current)).

### Concealment mechanism {16b}

Participants are randomised after eligibility criteria are confirmed.Participants will be randomised to either the RELAX intervention alongside UC or receive UC alone. The complete randomisation list sequence will be concealed from the investigators including the Chief Investigator (CI) and statisticians. The research team become aware of a single individual arm allocation at the point of randomisation by viewing the participant’s record on the password protected researcher’s interface platform. The researcher’s interface is built and hosted by Avegen and is used by the researchers to enrol and monitor participant progress. It is also where participant entered assessments and session data can be viewed and edited if verified to be missing or incorrect by the participant. Any edits of this sort are time stamped with the researchers’ initials for transparency. The researcher’s interface also holds researcher data, e.g. participant contact and payment logs.

Participants will be made aware of their randomisation arm via a message on the platform after submitting their baseline assessment. When viewing the Dashboard, it will either present the RELAX sessions and assessments (RELAX + usual care arm) or just the assessments (Usual care arm). Each session or assessment is available and unlocked once a participant has reached the applicable session or assessment timepoint.

### Implementation {16c}

The bespoke RELAX randomisation algorithm has been built into the platform by Avegen’s data team and programmers. Stratified randomisation will be carried out via the RELAX platform by Avegen in a 1:1 ratio using randomly permuted blocks with varying block sizes. Stratification is by recruitment site (GSTT vs KCH vs ESTH vs MCHT vs KCL), parity (0 vs not 0), and pregnancy complications (no pregnancy complications vs pregnancy complications). The information needed to classify participants based on the stratification variables will be gathered via the screening questionnaire (and verified on the study outline call). This information will be entered by the researchers into the researcher’s interface, providing the information required for randomisation.

## Assignment of interventions: blinding

### Who will be blinded {17a}

The CI, Co-Investigators and Principal Investigators at each study site will be unblinded at the individual level but will remain fully blinded at the group level (will not see data summarised by arm) until they review the statistical report, at which point they will become fully unblinded. The senior statistician will not have access to the randomisation list or the database platform at any point in the trial and will remain fully blinded until review of the first draft of the statistical reports for checking, when they will become fully unblinded. The trial statistician will be fully blinded until approval of the statistical analysis plan, after which they will be fully unblinded so they can inspect and utilise platform usage/intervention-related data. The Trial Coordinator, Research Assistant(s), Research Midwife, and any other members of the study team (e.g. students/volunteers working on the study) will be unblinded at the individual level only. The only individuals that will be able to summarise/see data by arm prior to the review of the statistical report are the trial statistician and the members of the Data Monitoring and Ethics Committee (DMEC), with the latter remaining partially blinded (i.e. arms shown as A/B) at the group level. The Trial Steering Committee (TSC) will remain blinded at both group and individual levels throughout the trial. As clinical staff in each investigation site are unblinded at the individual level, they will be able to reveal an individual’s allocation if needed.

### Procedure for unblinding if needed {17b}

If study team members are unintentionally unblinded, this will be recorded as a protocol deviation. Implications for analysis will be discussed with the DMEC. Any deviations will be documented and reported to the relevant authorities (e.g. Medicines and Healthcare products Regulatory Agency (MHRA)) as soon as possible.

As participants are not blinded, there is no need to have a process for unblinding participants.

## Data collection and management

### Plans for assessment and collection of outcomes {18a}

Participant sessions and assessments will be completed online, with data inputted by the participant into the RELAX platform built and hosted by Avegen. Each participant will have their own unique ID and enter the platform using their email address and their own set password. The platform therefore delivers the intervention and collects the data, which is saved into the database hosted by Amazon Web Services (AWS).

Participants self-complete all questionnaires for the assessments online. There is, however, the option for participants’ data to be collected from the participant in a telephone call with the researcher. In such instances, the researcher will input the participant’s responses via the researcher’s interface. This can be done with approval of the CI and in cases where a participant is unable to complete the assessments online themselves and require this additional support.

### Plans to promote participant retention and complete follow-up {18b}

Participants will receive £25 in online vouchers for each of the four assessments they complete (which is routine practice in mental health trials) to compensate for their time and to maximise data collection rate. Furthermore, there will be a prize draw for anyone who completes the screening questionnaire with the chance to win a £50 voucher. Members of our PPIE advisory group have favoured the idea of running a prize draw, which will occur every 3 months throughout the recruitment period.

Participants will receive email notifications to complete their assessments 1 week prior to their assessment opening, and on the day of assessment opening through the online RELAX platform. Automatic reminders will be sent a week after an assessment has been opened if it has not been completed. Researchers will also prompt participants at least three times after an assessment is opened if it remains incomplete. Prompts may be via telephone, text message or email. T1 and T2 assessments remain open for 3 weeks, and T3 assessments for 8 weeks following the assessment’s due date. Participants who withdraw or are lost to follow-up will not be replaced.

### Data management {19}

Online responses will be collected using the RELAX platform. All data collected will be stored using Amazon Web Services (AWS). Any other screening data will be collected by the study team and uploaded to the researcher interface, feeding into the database on AWS.

To ensure data quality, data checking will be carried out throughout the trial. Data anomalies will be queried by the trial statistician and corrected by the team where possible (as much of the source data will be entered directly into the Avegen platform by the participants) prior to statistical analysis.

### Confidentiality {27}

All personal data collected during screening and recruitment will be stored as password-protected files on KCL secure servers (or physical paper copy documents or items such as audio recorders in locked storage on KCL property). NHS secure servers will store information held by the Research Midwife, such as recruitment logs and if a person has consented to be contacted about the project by the research team. Personal data of participants and potential participants will also be stored on the RELAX platform and researcher interface on Avegen’s secure database. Only researchers directly involved in the study will have access to the data. Participants’ personal data will be retained for up to a year following the end of the clinical trial. Personal data of potential participants will be retained until the end of the recruitment period. During the trial, all data will be pseudonymised using unique identification numbers and stored without identifying information (names, email addresses, phone numbers). Once the trial is completed, data will be stored anonymously and securely for 12 years.

### Plans for collection, laboratory evaluation and storage of biological specimens for genetic or molecular analysis in this trial/future use {33}

Not applicable as no biological samples are to be collected.

## Statistical methods

### Statistical methods for primary and secondary outcomes {20a}

A statistical analysis plan (SAP) has been drafted by the senior statistician and approved by the DMEC and TSC. The SAP is available on request from the corresponding author and trial statisticians.

The primary population for analysis will be the intention to treat (ITT), defined as all randomised participants, analysed in the arms they were randomised to, regardless of which intervention they received.

#### Analysis of primary and secondary clinical outcomes

The mean difference in GAD-7 between arms at 8 weeks post-randomisation will be estimated using a mixed-effects linear analysis of covariance (ANCOVA) model with repeated measures and a random intercept at the participant level. We will include as dependent variables the GAD-7 scores at 4 weeks, 8 weeks (primary outcome) and 36 weeks post-randomisation. We will also adjust for trial arm, time, baseline anxiety (GAD-7), weeks of gestation at baseline (a pre-specified baseline variable), the three randomisation stratification variables, and an interaction term for trial arm and time to allow effects to differ over time and be extracted at the different time points. Furthermore, if any baseline variables are found to predict missing outcome data, we will include them as covariates in our model. The mean differences between arms for the GAD-7 at 36 weeks secondary outcome will be estimated using the model described for the primary outcome, with the mean differences between arms for the other secondary outcomes estimated using similar models to those described for the primary outcome.

### Interim analyses {21b}

There are no interim analyses planned.

### Methods for additional analyses (e.G. Subgroup analyses) {20b}

#### Analysis of the mechanistic outcome/mediation analysis

To estimate the extent to which the intention to treat RELAX vs UC intervention effect on anxiety (GAD-7) at 8 weeks (primary) or 36 weeks (secondary) is mediated by interpretation bias (measured using the Recognition Test) at 4 weeks, we will use linear structural equation models estimated using full information maximum likelihood estimation to fit mediation models. We will fit two separate structural equation models for the 8-week and 36-week GAD-7 outcome measures, each fitting two equations: one with the 4-week interpretation bias (RT) mediator as the dependent variable and the other with the GAD-7 outcome as the dependent variable with the RT mediator as an independent variable. Both mediator and outcome models will have arm as a binary independent variable, include the three randomisation stratification factors and the weeks of gestation at baseline variable as independent variables, and will include baseline GAD-7 and interpretation bias (RT) measures as independent variables.

The indirect (or mediated) effect will be calculated in each case by multiplying the estimate of the effect of the intervention on the interpretation bias mediator by the estimate of the effect of interpretation bias on the GAD-7 outcome. For this indirect effect, we will present the percentile bootstrap confidence interval from bootstrapping with 1000 repetitions [51]. We will also present this proportion of the total effect that mediated for each model, calculated as the indirect effect estimate divided by the total effect estimate, multiplied by 100.

#### Subgroup analysis/moderation of treatment effect

The study is not formally powered for subgroup analyses, but we will investigate in an exploratory fashion whether 8-week anxiety (as measured by the GAD-7) intervention effects differ by levels of baseline anxiety, pregnancy complications yes/no at baseline and pre-existing physical health conditions yes/no at baseline. The two-way trial arm by time point interaction terms will be extended to three-way trial arm by time point by baseline subgroup variable terms added to the main analysis model for the primary anxiety outcome (see section “Statistical methods for primary and secondary outcomes {20a}”). If these interaction terms are statistically significant with respect to the subgroup variable of interest, we will present intervention versus control estimates at 8-weeks post-randomisation by baseline anxiety, pregnancy complications, and/or pre-existing physical health conditions levels. This analysis will be clearly stated as exploratory in the primary paper/report and will be interpreted accordingly.

### Methods in analysis to handle protocol non-adherence and any statistical methods to handle missing data {20c}

#### Complier average causal effect analysis

We have also specified a Complier Average Causal Effect (CACE) analysis for the 8-week primary GAD-7 outcome to estimate intervention effects in those who completed all 30 trials in a session for at least 10 out of the 12 RELAX sessions. We will use an instrumental variable approach using the stata command `xtivreg` to handle the longitudinal data, and generally include the same covariates used for the main ITT analysis of the primary GAD-7 outcome.

#### Missing data

Where available we will use missing value guidance provided for scales. Where this is not available, we will prorate missing items only when there are no more than 20% missing items by replacing the missing item values with the mean value of the complete items for each individual. Missing baseline data will be simply imputed [52]. Missing outcome data will mainly be dealt with using maximum likelihood methods to fit the mixed models and including baseline variables that predict missing outcome data (to make the missing at random assumption more plausible). We will consider performing multiple imputation for primary and secondary outcomes only if there are post-randomisation variables that are predictive of missingness for these measures, and the proportion of participants with missing values for any of the primary or secondary outcome variables is equal or greater to 10% [53].

### Plans to give access to the full protocol, participant-level data and statistical code {31c}

The datasets generated during and/or analysed during the current study will be stored in the King’s Open Research Data System (KORDS), which is a repository that allows data sets to be shared openly [54].

Any data shared will be pseudonymised final datasets made available following publication of the trial papers, as agreed by the Trial Management Group nearer to the time of the deposit.

The local Research and Development office has reviewed these plans and advised that the participant consent is currently appropriate for this data sharing, i.e. with the participant clause in the consent form: “I understand that the research team may use my data for future research and that my data may be shared anonymously with other researchers”. There are no plans to share the statistical code or full protocol.

## Oversight and monitoring

### Composition of the coordinating centre and trial steering committee {5d}

The RELAX research team (i.e. Trial Coordinator, Research Assistant(s), Research Midwife and trained and supported students/volunteers working on the project) are responsible for the day to day running of the trial and participant contact. This may be to screen potential participants for eligibility, to contact participants as they progress through the RELAX intervention, to prompt participants to complete assessments, to complete post-intervention interviews or deal with queries relating to platform or risk issues. Oversight will be provided by the trial CI (a Clinical Psychologist). The team holds weekly team meetings to discuss any potential participant issues and to share wider trial updates with the CI. The RELAX research team meet monthly with the CI and the broader RELAX Trial Management Group, including NHS site Principal Investigators, statisticians, co-applications to provide broader trial updates and make key decisions.

The RELAX TSC will meet at least yearly to provide broader trial oversight. The TSC consists of trial specialists, statisticians, clinicians and the PPIE Lead for the study and three PPIE representatives, with a Charter of membership developed and signed.

#### Patient and public involvement and engagement

We have worked closely with PPIE representatives from the conception of this research. We will continue to work closely with our PPIE group to ensure that the needs of the ultimate beneficiaries of the research (pregnant women/service users) remain central to the project. Thus, we will ensure that the study:

- Develops materials pertinent to pregnant women’s day to day concerns,
- Identifies multiple scenarios that trigger RNT and anxiety in a broad range of pregnant women,
- Has community and maternity service users’ feedback on proposed adaptations to the intervention and trial plans,
- Has community and maternity service user advice on ways of engaging with the target audience and maximising participation rates, particularly focusing on issues of diversity and inclusion, and
- Promotes and develops good practice in PPIE, reporting our methods and learning.

Study findings will be communicated to maternity services, charities, support networks, and community and professional organisations, ensuring that communication messages, language and images are acceptable, relevant and appealing to target audiences and service users from different communities, and informed by the advice of named third sector organisations and advocacy groups (including King’s College Denmark Hill Maternity and Neonatal Voices Partnership (MNVP), Maternal Mental Health Alliance, National Maternity Voices). We will hold an online learning event, write a blog for public and professional health service audiences, and use social media to share and discuss the findings. Further information is publicly available at the NIHR Applied Research Collaboration (ARC) South London website [55].

### Composition of the data monitoring committee, its role and reporting structure {21a}

The RELAX DMEC is set up to be an advisory group to the TSC. As there is no planned interim analysis, the DMEC will monitor the trial and communicate any concerns to the TSC, who can decide to terminate the trial prematurely if necessary. The DMEC consist of trial specialists, statisticians and clinicians, and a Charter of membership has been developed and signed.

### Adverse event reporting and harms {22}

All adverse events and serious adverse events (SAE) for participants (and their infants) will be recorded on the RELAX platform. Clinical symptoms will be documented and accompanied with a simple, brief description of the event, including dates as appropriate, as well as an assessment of the event’s severity and relatedness to the intervention, initially completed by the researcher and signed off by the CI. If it is in the best interest of the participant or clinical team managing a participant’s maternity care, the study team will inform the clinical team of any AEs that a participant experiences. For participants linked to one of the four participating NHS sites, this will be done promptly (usually on the same day), where the site PI will have access to review the event details on the platform researcher’s interface. SAE and adverse device effects will be reported to the sponsor within 24 h of the researcher becoming aware of the event. Relevant regulatory authorities and the platform manufacturer will also be made aware of the event. The trial protocol includes a detailed list of expected pregnancy-related SAEs, e.g. hospital admission for active labour, that are documented but exempt from the same level of sponsor and regulatory authority reporting.

### Frequency and plans for auditing trial conduct {23}

The investigators and the institutions will permit trial-related monitoring, audits, REC review and regulatory inspections (where appropriate) by providing direct access to source data and other relevant documents.

### Plans for communicating important protocol amendments to relevant parties (e.G. Trial participants, ethical committees) {25}

In the event of any amendments, the Sponsor’s Research and Development office and regulatory ethics boards will be notified. In addition, where appropriate, the MHRA will also be informed. Prior consultation will be undertaken with trial PPIE, TMG, TSC and DMEC committees, as appropriate, as well as with the funder (NIHR).

### Dissemination plans {31a}

The planned dissemination route is to further develop a sustainable model for RELAX. This will cover updates to software, materials and technical support (OS updates and upgrades), as well as web-platform hosting. RELAX could potentially be licenced for use by NHS services and third sector organisations who support pregnant women. Implementation plans could be assessed during a subsequent effectiveness trial.

We will send all participants a summary of the study findings using lay language after data analysis has been completed. We will present the study findings at conferences, hold dissemination workshops for stakeholders and our participating NHS sites. We will also produce policy lab briefings, working together with the KCL Policy Institute and our PPIE group.

We will produce a publication on the role, practice and impact of PPIE in the study, publish trial data in high-impact journals and publicise our findings via social and published media to disseminate the clinical results to a broader audience. Our PPIE group will work with us to develop the dissemination plan.

## Discussion

The RELAX Study is a parallel two-arm randomised controlled trial testing an online interpretation training intervention to evaluate whether at-risk pregnant women who complete RELAX alongside usual care report less perinatal anxiety before and after birth than those who receive usual care only. RELAX is based on an evidence-based intervention previously shown to reduce anxiety in non-pregnant populations [25–27], which we have adapted and tailored for a pregnant sample. Specifically, it is designed for pregnant women who experience high levels of RNT, an established risk factor for later anxiety. On the basis that RNT is maintained by a negative interpretation bias, RELAX aims to reduce the tendency to make negative interpretations (i.e. the theorised mechanism of the intervention) in order to reduce RNT, in turn resulting in lower levels of anxiety symptoms. To our knowledge, RELAX is the only pregnancy-tailored online intervention specifically aimed at targeting RNT.

This research addresses the need for targeted early interventions for perinatal anxiety, which feature in the NHS Long Term Plan. It is well-established in non-perinatal populations that interventions which target individuals who are identified as being at-risk of psychological problems are more effective than non-targeted universal approaches [56]. Accordingly, the development of a low-intensity (self-help), highly accessible, cost-effective intervention to address perinatal anxiety is an exciting prospect.

We have included PPIE contributors with lived experience of perinatal anxiety in the design and development of the trial. Our PPIE members have indicated that interventions for perinatal anxiety delivered online would be a feasible and welcomed way to engage at-risk pregnant women. This population typically has multiple responsibilities, rendering a home-based intervention that can be completed at a time convenient for them as having great appeal. The PPIE members have also been involved in the development of the scenarios used in the intervention, alongside contributing to the bespoke RELAX platform design and testing the platform during the usability testing phase, thus ensuring that the intervention platform was built, tested and endorsed by the intended service user group. The trial team involves experts and clinicians from psychology, midwifery and biostatistics, as well as an expert PPIE researcher. Each team member brings their unique perspectives to the project, leading to meaningful muti-disciplinary research and effective public involvement from a broad and highly motivated group.

In sum, this trial will establish whether an intervention for pregnant women with high levels of RNT that does not require delivery by a mental health specialist can prevent escalating anxiety in the perinatal period. Should the trial demonstrate that RELAX is efficacious, an effectiveness trial to aid implementation could be conducted. Moreover, evidence of efficacy has the potential to open other avenues for future research, e.g. investigations of whether there are longer-term, sustained positive impacts of the intervention post birth (i.e. in the “fourth” trimester), when the additional changes, challenges and worries of motherhood become apparent. In addition to scaling up the intervention for use in pregnancy, there is scope for it to be translated into languages other than English, and developed for other groups, such as partners of pregnant women and birthing people, and pregnant women who already report high levels of anxiety and/ or psychiatric diagnoses.

## Trial status

The current protocol is version 1.9 dated 18th January 2024. Participant recruitment began on 8th June 2023 and was expected to end 30th June 2024. We anticipate that recruitment will now finish by the end of July 2024.

## Data Availability

Please refer to our earlier section on “Plans to give access to the full protocol, participant-level data and statistical code”.

## Abbreviations

ANCOVA: Analysis of covariance
ARC: NIHR Applied Research Collaboration
AWS: Amazon Web Services
BRC: Biomedical Research Centre
CACE: Complier Average Causal Effect
CBM: Cognitive bias modification
CBM-I: Cognitive bias modification for interpretation
CBT: Cognitive behavioural therapy
CI: Chief Investigator
CIS-R: Clinical interview schedule revised
DMEC: Data Monitoring and Ethics Committee
EME: Efficacy and Mechanism Evaluation
EPDS: Edinburgh Postnatal Depression Scale
ESTH: Epsom and St Helier University Hospitals NHS Trust
GAD: Generalised anxiety disorder
GAD-7: Generalised Anxiety Disorder Questionnaire
GP: General Practitioner
GSTT: Guy’s and St Thomas’ NHS Foundation Trust
ID: Identifier
ITT: Intention to treat
KCH: King’s College Hospital NHS Foundation Trust
KCL: King’s College London
KORDS: King’s Open Research Data System
MCHT: Mid Cheshire Hospitals NHS Foundation Trust
MHRA: Medicines and Healthcare products Regulatory Agency
MNVP: Maternity and Neonatal Voices Partnership
MRC: Medical Research Council
NCT: National Childbirth Trust
NHS: National Health Service
NIHR: National Institute for Health and Care Research
OS: Operating System
PASS: Perinatal Anxiety Screening Scale
PHQ-9: Patient Health Questionnaire
PIC: Participant Identification Centre
PPIE: Patient and Public Involvement and Engagement
PSWQ: Penn State Worry Questionnaire
REC: Research Ethics Committee
RELAX: REducing Levels of AnXiety
RT: Recognition Test
RTQ-10: Repetitive Thinking Questionnaire
SAE: Serious adverse event
SAP: Statistical Analysis Plan
SMS: Short Messaging Service
TMG: Trial Management Group
TSC: Trial Steering Committee
RNT: Repetitive negative thinking
UC: Usual Care
UK: United Kingdom
WSAS: Work and Social Adjustment Scale

## Footnotes

1 Should a participant wish to be screened again they will be sent a link to complete a new screening assessment.

2 Due to an error in Session 9, 87% of scenarios were presented in an ambiguous form.
